# Inflammatory potential of diet and bone mineral density in a senior Mediterranean population: a cross-sectional analysis of PREDIMED-Plus study

**DOI:** 10.1007/s00394-021-02751-5

**Published:** 2021-11-29

**Authors:** Jesús F. García-Gavilán, Indira Paz-Graniel, Nancy Babio, Dora Romaguera, Jose Alfredo Martínez, Vicente Martin, María Ángeles Martínez, Jadwiga Konieczna, Miguel Ruiz-Canela, José Antonio de Paz Fernandez, Albert Goday, Miguel Ángel Martínez-González, Mònica Bulló, Jordi Salas-Salvadó

**Affiliations:** 1 Universitat Rovira i Virgili, Department of Biochemistry and Biotechnology, Human Nutrition Unit, C/Sant Llorenç 21, 43201 Reus, Tarragona Spain; 2 Institut d’Investigació Sanitària Pere Virgili (IISPV), Reus, Spain; 3 CIBER Physiology of Obesity and Nutrition (CIBEROBN), Carlos III Health Institute, Madrid, Spain; 4 Research Group on Nutritional Epidemiology and Cardiovascular Physiopathology (NUTRECOR), Health Research Institute of the Balearic Islands (IdISBa), University Hospital Son Espases (HUSE), Palma de Mallorca, Spain; 5 Department of Nutrition, Food Sciences, and Physiology, Center for Nutrition Research, University of Navarra, Pamplona, Spain; 6 Precision Nutrition and Cardiometabolic Health Program, IMDEA Food, CEI UAM + CSIC, Madrid, Spain; 7 Institute of Biomedicine (IBIOMED), University of León, León, Spain; 8 CIBER de Epidemiología y Salud Pública (CIBERESP), Instituto de Salud Carlos III, Madrid, Spain; 9 Unit of Cardiovascular Risk and Nutrition, Hospital del Mar Medical Research Institute (IMIM), Barcelona, Spain; 10 Department of Preventive Medicine and Public Health, Navarra Health Research Institute (IDISNA), University of Navarra, Pamplona, Spain; 11 Department of Nutrition, Harvard TH Chan School of Public Health, Boston, MA USA; 12 Departament de Medicina, Univeristat Autonoma de Barcelona, Barcelona, Spain

**Keywords:** Diet, Bone mineral density, Inflammation, Osteoporosis

## Abstract

**Purpose:**

Inflammation could play a key role in tissue damage and bone metabolism. The modified dietary inflammatory score (M-DIS) is a validated tool to estimate the inflammatory potential of the diet. In the present study, we evaluate the associations between the M-DIS and bone mineral density (BMD) in a senior Mediterranean population with overweight/obesity and metabolic syndrome.

**Methods:**

Baseline cross-sectional association between the M-DIS and bone mineral density was assessed in 1134 participants of the multicenter PREDIMED-Plus trial (aged 55–75 with overweight/obesity and metabolic syndrome). BMD was measured using Dual-energy X-ray Absorptiometry scans and participants answered a food frequency questionnaire to determine the M-DIS. BMD was categorized as low BMD when *T* score was equal or lower than -1 and normal BMD in another case. Associations between BMD and M-DIS were evaluated by using linear and logistic regressions adjusted by other co-variates.

**Results:**

Participants in the top tertile of the M-DIS had a lower BMD at total femur [β (95% CI) − 0.02 (− 0.04, − 0.01)], trochanter areas [β (95% CI) − 0.03 (− 0.05, − 0.01)] and lumbar spine area [β (95% CI) − 0.03 (− 0.07, 0.01)] (but in the last case, measures were less precise and hence not statistically significant) compared to those in the lower M-DIS tertile. Multiple logistic regression analyses showed that the odds of the total femur and femoral trochanter osteopenia/osteoporosis were higher in participants in the top tertile compared to those in the lowest tertile of M-DIS [OR (95% CI) 1.71 (1.12, 2.64), *P* for trend 0.015; 2.02 (1.29, 3.21), *P* for trend 0.002, respectively].

**Conclusion:**

A high pro-inflammatory diet, measured by the M-DIS, is associated with lower BMD in a senior Mediterranean population with metabolic syndrome.

**Supplementary Information:**

The online version contains supplementary material available at 10.1007/s00394-021-02751-5.

## Introduction

Osteoporosis is an age-related chronic disease characterized by the loss of bone mass and trabecular alterations that decline bone strength and increases the risk of fractures. These changes are influenced by non-modifiable factors like genetics and age-related hormone changes, and by modifiable factors related to current lifestyles such as the quality of diet, physical activity, sedentary behaviors, and smoking habits [1, 2].

Aging is the strongest risk factor for the development of musculoskeletal disorders and promotes fat mass accumulation, sarcopenic obesity, bone loss, inflammation, and oxidative stress [3, 4]. Several epidemiological studies have associated systemic inflammation with an increased risk of developing non-communicable chronic diseases, especially obesity, Type 2 Diabetes (T2D), and cardiovascular diseases, but also with osteoporosis, frailty, and sarcopenia [5–8]. In this regard, it has been suggested that inflammation could play a key role in tissue damage and bone metabolism [9]. Some pro-inflammatory cytokines, such as Tumor Necrosis Factor-alpha (TNF-α) or interleukin 6 (IL-6), may exert an inhibitory effect on osteoprotegerin (OPG) increasing Receptor Activator for Nuclear Factor κ B Ligand (RANKL) concentrations, and consequently, increasing the osteoclastic activity, bone resorption and the risk of osteoporosis incidence [10, 11].

In addition, diet plays a role in the modulation of inflammation and may have a relevant role in the prevention of osteoporosis. Healthy dietary patterns, such as Mediterranean diet (MedDiet), characterized by their high content in fruit, vegetables, whole grains, and fish and that contribute to the intake of nutrients like fiber, omega-3, monounsaturated fatty acids, or vitamin D have been demonstrated to have anti-inflammatory properties and to reduce the bone reabsorption process [12, 13]. Contrary, other studies have linked a higher adherence to pro-inflammatory dietary patterns distinguished by its content in processed food, fats, and red meat with lower bone mineral density (BMD) and increased peripheral inflammation [12–15].

In this context, the modified dietary inflammatory score (M-DIS) is a validated tool to estimate the inflammatory potential of the diet [16]. Previous studies have shown that higher M-DIS scores are significantly associated with higher circulating inflammatory markers and increased risk of metabolic alterations and chronic diseases such as obesity, metabolic syndrome, T2D, cardiovascular disease, and osteoporosis [16–20]. A recent meta-analysis of epidemiological studies has reported an inverse association between M-DIS and BMD at the lumbar spine and total hip in both men and women, but not at the femoral neck, along with an increased risk of osteoporosis and fractures in those individuals with higher M-DIS scores [21]. Similarly, in a cross-sectional epidemiological study, an inverse correlation between M-DIS and total BMD was reported in a subsample of 121 postmenopausal women [22]. However, the high heterogeneity among the studies included in this meta-analysis, mainly conducted in postmenopausal women with Western diet, the lack of studies conducted in the Mediterranean populations, and other recent publications which did not find associations between these parameters, do necessary to value the usefulness of this score in other contexts where the dietary pattern is considered anti-inflammatory [23, 24]. Therefore, new prospective studies involving different populations, and pooled by sex, are needed to increase the generalization of the result, and the level of evidence of the possible associations between diet, inflammation, and bone status or metabolism.

In the present study, we evaluated the associations between the M-DIS score and bone mineral density in a senior Mediterranean population with overweight/obesity and metabolic syndrome.

## Materials and methods

This is a cross-sectional analysis conducted in a subset of 1134 participants from the PREDIMED-Plus study, a multicenter, randomized, and parallel-group clinical trial including 6874 women and men, aged 55–75 with overweight/obesity [body mass index (BMI) between 27 and 40 kg/m^2^] and metabolic syndrome defined by the updated harmonized criteria of the International Diabetes Federation, the American Heart Association, and the National Heart Association [25]. Participants with severe chronic diseases, drug or alcohol addiction, or allergy to MedDiet food were excluded from the study. For the present study, we have included only randomized individuals with DXA measurements participating in a body composition substudy from 4 of the 23 PREDIMED-Plus recruiting centers: Reus (UNH-URV), Mallorca (Hospital Son Espases/IDISBA), Pamplona (IDISNA) and León (IBIOMED). Participants included in the present analysis with DEXA measurements did not differ from the rest of the participants enrolled in the PREDIMED-Plus trial in terms of age, sex, BMI, and prevalence of obesity and T2D (*P* > 0.05 for all comparisons).

Detailed protocol and study information was previously published [26] and is available at http://predimedplus.com. Both the protocol and procedures were implemented following the ethical standards of the Declaration of Helsinki and approved by the institutional ethics review boards of each study center (Ref: 13-07-25/7proj2). In addition, all participants provided written informed consent. The PREDIMED-Plus study was registered at http://www.isrctn.com/ (ISRCTN89898870).

## Bone assessment

An X-ray Bone Densitometer (DXA) (DXA Lunar Prodigy Primo and Lunar iDXA; GE Healthcare, Madison, WI) was used to assess bone mineral density (BMD) (g/cm^2^). For this work, we used the following bone areas for their clinical relevance: the total femur (TF), lumbar spine (from L1 to L4) (LS), and trochanter (TR). The BMD at the femoral area was measured on the non-dominant side. The *T* score of these three measurements (TF, LS, and TR) were calculated using the reference values for the Spanish adult population included in the DXA software considering sex, age, weight, and height of the reference population.

## Dietary assessment

Dietary intake was estimated using a validated semi-quantitative food frequency questionnaire (FFQ) composed of 143 items [27]. Energy and nutrient intakes were calculated using Spanish nutritional food composition tables [28, 29]. The method used to estimate the M-DIS scores have been previously described and published [16]. In short, the M-DIS is a score calculated using previously published articles that assessed the effect of 45 food, nutrients (macro and micro), and compounds on several inflammatory biomarkers. Each parameter was scored according to its influence on these inflammatory biomarkers (+ 1 if the parameter increased the inflammatory biomarkers, 0 if it did not have any effect on them, or − 1 if it decreased them). To estimate the individual M-DIS score, the intake of every food item was standardized using the means and SD of each food/nutrient item [16]. The final score is the sum of all its components for each participant. This allows us to classify the participant's diet as an anti-inflammatory (negative values) or pro-inflammatory (positive values) diet. Like previous studies [17], we used the following 32 nutrient/food parameters were used to compute the M-DIS score available in the PREDIMED-Plus study: caffeine (g), alcohol (g), vitamin B1 (mg), vitamin B2 (mg), vitamin B3 (mg), vitamin B6 (mg), vitamin B12 (μg), vitamin A (RE), vitamin C (mg), vitamin E (mg), vitamin D (μg), carbohydrates (g), protein (g), cholesterol (mg), total fatty acids (g), monounsaturated fatty acids (g), polyunsaturated fatty acids (g), saturated fatty acids (g), trans-fatty acids (g), energy intake (kcal), fiber (g), folic acid (μg), garlic (g), iron (mg), magnesium (mg), selenium (μg), zinc (mg), n-3 fatty acids (g), n-6 fatty acids (g), beta-carotene (μg), onions (g), and tea (g).

## Other variables measurements

Trained dietitians collected information about lifestyle habits, health status conditions, and medication used. Leisure-time physical activity was evaluated with the validated Spanish version of the Minnesota Leisure-Time Physical Activity Questionnaire [30]. BMI was calculated as weight (kg) divided by the square of height (m^2^). Both weight and height were measured with calibrated scales and light clothes.

## Statistical analysis

Participants were categorized by tertiles of the M-DIS score. Participant characteristics were described according to these tertiles as mean ± standard deviations (SD) when variables were quantitative or percentages (*n*) when variables were categorical. Differences between tertiles were tested using analysis of variance (ANOVA) or chi-square test, respectively. As total Osteoporosis cases were insufficient to do a statistical analysis considering the typical three categories of BMD state (normal state, Osteopenia state, and Osteoporosis state) without producing a bias, we assessed the association between the M-DIS score and Osteopenia/Osteoporosis status using a dichotomic variable (referred to as “low BMD status”) that was made considering the BMD *T* scores and a modification of the *T* score cut-offs established by the World Health Organization (WHO) for each area [2]: low BMD status (1) when the *T* score of TF, TR, or LS was equal or lower than − 1; normal BMD status (0) when *T* score values were higher than − 1.

Several models were used to evaluate the association between M-DIS and BMD. Analysis of covariance (ANCOVA) was used to compare differences between tertiles of M-DIS of BMD in TF, TR, and LS. All models were adjusted for sex (woman/man), the prevalence of T2D (yes/no), age (years), BMI (kg/m2), educational level (illiterate/primary education, secondary education, and academic/graduate), smoking status (never/current/former), physical activity (METs/day), use of insulin (yes or no), use of oral antidiabetic drugs (yes or no), use of oral anticoagulants drugs (yes or no), use of calcium and vitamin D supplements (yes or no), and use of estrogens (yes or no), recruitment center and total energy intake (kcal/day). The assumptions of the ANCOVA models were checked using visual or quantitative methods. All graphs and tests yielded models that met the independence of observations, homogeneity of variance, and normality of residuals criteria. Additionally, the Tukey test was used to make multiple comparisons between M-DIS tertiles.

Linear and logistic regression models were fitted to assess the associations between M-DIS and BMD in TF, TR, and LS (linear regression models) or low BMD status (logistic regression). For these analyses, we used tertiles of M-DIS in both linear and logistic models, considering the first tertile (low M-DIS) as the reference. Multivariable models were adjusted for sex (woman/man), the prevalence of T2D (yes/no), age (years), BMI (kg/m^2^), educational level (illiterate/primary education, secondary education, and academic/graduate), smoking status (never/current/former), physical activity (METs/day), use of insulin (yes or no), use of oral antidiabetic drugs (yes or no), use of oral anticoagulants drugs (yes or no), use of calcium and vitamin D supplements (yes or no), and use of estrogens (yes or no), recruitment center and total energy intake (kcal/day). Like the ANCOVA models, assumptions of the linear and logistic regressions were checked using visual or quantitative methods. All graphs and tests yielded models that met the linearity, independence of errors, homoscedasticity, and normality of residuals criteria.

Stratified logistic regression analyses were conducted in TF, TR, and LS as sensitivity analyses dividing participants by age (< 70 years/ ≥ 70 years), sex, BMI (< 30 kg/m^2^/ ≥ 30 kg/m^2^), and T2D. Models were adjusted by the same variables used previously in the main analyses excluding the, respectively, stratified variable.

The P for trend in linear and logistic models was assessed modeling the M-DIS score as a continuous variable. Interactions with sex, T2D, and BMI were evaluated using the likelihood ratio test including the interaction product term as covariables. Because there was no effect significant modifications (*P* > 0.05), all the analyses were performed with the complete study population.

For these analyses, we used the official PREDIMED-Plus database updated on 17 September 2018. It was considered statistically significant all *P* values < 0.05. The present statistical analyses were performed with the R software v3.6.1 (www.r-project.org) (R Development Core Team, 2012).

## Results

The total sample of participants included in this analysis (48% women) was 65 ± 5 years old, had a mean BMI of 32.6 ± 3.4 kg/m^2^, reported an average physical activity expenditure of 380 ± 340 MET/day, and an average energy intake of 2469 ± 592 kcal/day. A total of 41% of participants were never smokers, and the prevalence of T2D was 22%. The mean M-DIS of the full sample was -3.87 ± 5.13.

The total number of participants with TF measurements were 1105, for TR 1130, and for LS 985. The distribution of M-DIS was similar between BMD measurements. Compared to participants in the higher BMD tertile, those in the lower tertile showed a mean M-DIS of − 9.61 ± 3.39 for TF, − 9.62 ± 3.50 for TR, and − 9.50 ± 3.55 for LS, while the higher tertiles showed a mean M-DIS of 1.44 ± 2.21 for TF, 1.41 ± 2.39 for TR, and 1.50 ± 2.35 for LS.

Table 1 shows the baseline characteristics of the studied population by tertiles of the M-DIS score. Participants in the lowest M-DIS tertile (highest anti-inflammatory capacity) had higher physical activity and adherence to the MedDiet than those in the top tertile. Education was also significantly different across M-DIS tertiles. No differences across M-DIS tertiles were observed about age, sex, BMI, smoking status, prevalence of T2D, prevalence of osteoporosis, and medication use.

**Table 1:** Baseline characteristics of PREDIMED Plus participants by tertiles of M-DIS

	Tertile 1 (*n* = 368)	Tertile 2 (*n* = 368)	Tertile 3 (*n* = 368)	*P* value
M-DIS	− 9.61 ± 3.39	− 3.36 ± 1.18	1.44 ± 2.21	
Age, years	65.43 ± 4.83	64.86 ± 4.94	64.90 ± 5.19	0.229
Sex, women % (*n*)	45.40 (167)	46.20 (170)	51.60 (190)	0.182
BMI (kg/m^2^)	32.57 ± 3.46	32.53 ± 3.27	32.82 ± 3.38	0.448
Physical activity, METs/d	438.19 ± 376.57	377.60 ± 339.40	326.83 ± 289.02	< 0.001
Smoker, % (*n*)				0.089
Never	41.60 (153)	39.70 (146)	43.20 (159)	
Current	10.10 (37)	12.50 (46)	14.70 (54)	
Former	48.30 (178)	47.80 (176)	42.10 (155)	
Education, %, (*n*)				0.043
Illiterate/primary	52.40 (193)	48.70 (189)	54.40 (200)	
Secondary	25.00 (92)	34.20 (126)	29.60 (109)	
Academic/graduate	22.60 (83)	17.10 (63)	16.00 (59)	
Type 2 diabetes, % (*n*)	22.80 (84)	20.40 (75)	22.30 (82)	0.701
Osteoporosis, % (*n*)	1.10 (4)	0.30 (1)	1.40 (5)	0.269
Dyslipidemia, % (*n*)	71.74 (264)	66.85 (246)	68.21 (251)	0.388
Total energy intake, kcal/d	2839.50 ± 555.80	2450.50 ± 514.40	2108.60 ± 473.20	< 0.001
Adherence to MedDiet, p17	9.15 ± 2.55	8.45 ± 2.58	7.52 ± 2.31	< 0.001
Medication use, % (*n*)				
Insulin	3.80 (14)	2.99 (11)	2.72 (10)	0.681
Oral anticoagulants	20.11 (74)	18.21 (67)	19.29 (71)	0.806
Oral antidiabetic drugs	16.58 (61)	15.22 (56)	16.58 (61)	0.846
Estrogens	0.54 (2)	0.27 (1)	0.00 (0)	0.714
Calcium and vitamin D supplements	4.35 (16)	3.80 (14)	4.89 (18)	0.770

Values are presented as mean ± SD or percentage (*n*). *P* values were calculated with ANOVA test (quantitative variables) and Chi-square test (qualitative variables)

*M-DIS* modified dietary inflammatory score, *BMD* bone mineral density, *BMI* body mass index, *MedDiet* Mediterranean diet

Table 2 shows the beta-coefficients (*β*) and trends of associations between tertiles of M-DIS score and BMD. Compared to those in the lowest M-DIS tertile, participants in the highest tertile had a lower BMD at total femur [β (95% CI) − 0.02 (− 0.04, − 0.01)], trochanter [β (95% CI) − 0.03 (− 0.05, − 0.01)] and lumbar spine [β (95% CI) − 0.03 (− 0.07, 0.01)] after adjusting for potential confounders. However, the last area was less precise and hence not statistically significant. Moreover, the M-DIS showed a significant inverse association with BMD in the total femur (*P* value: 0.045), trochanter (*P* value: 0.012), and lumbar spine (*P* value: 0.039) areas. Higher M-DIS scores were also associated with lower BMD status in all three BMD measured sites after adjustment for potential confounders [(mean ± SD, T1 vs T3) TF: 1.04 ± 0.15 vs 1.02 ± 0.14, *P* value: < 0.001; TR: 0.88 ± 0.15 vs 0.85 ± 0.15, *P* value: < 0.001; LS: 1.19 ± 0.19 vs 1.16 ± 0.21; *P* value: 0.032].

**Table 2:** Associations of BMD by tertiles of M-DIS in the PREDIMED-Plus trial

	Tertile 1	Tertile 2	Tertile 3	*P* for trend
*n*	368	368	368	
Total femur, g/cm^2^	1.04 ± 0.15	1.02 ± 0.14†	1.02 ± 0.14†	< 0.001
Crude model	0 (reference)	− 0.02 (− 0.04, − 0.01)	− 0.02 (− 0.04, − 0.01)	0.047
Adjusted model*	0 (reference)	− 0.03 (− 0.04, − 0.01)	− 0.02 (− 0.04, − 0.01)	0.045
*n*	377	376	376	
Trochanter, g/cm^2^	0.88 ± 0.15	0.85 ± 0.14†	0.85 ± 0.15†	< 0.001
Crude model	0 (reference)	-0.03 (-0.05, -0.01)	− 0.03 (− 0.05, − 0.01)	0.002
Adjusted model*	0 (reference)	-0.03 (-0.04, -0.01)	− 0.03 (− 0.05, − 0.01)	0.011
*n*	328	328	328	
Lumbar spine L1–L4, g/cm^2^	1.19 ± 0.19	1.16 ± 0.18	1.16 ± 0.21	0.032
Crude model	0 (reference)	− 0.03 (− 0.06, 0.01)	− 0.03 (− 0.06, − 0.01)	0.042
Adjusted model*	0 (reference)	− 0.04 (− 0.06, − 0.01)	− 0.03 (− 0.07, 0.01)	0.039

ANCOVAs were used to compare covariate-adjusted mean of bone mineral density with tertiles of M-DIS. Linear regression models were used to evaluate the association between bone mineral density and tertiles of M-DIS. Results are expressed as β coefficients (95% CIs), and means ± SDs

*M-DIS* modified dietary inflammatory score

^†^*P* < 0.05 for comparisons between Tertile 2/Tertile 3 and Tertile 1 with Tukey Test

^*^Models adjusted for sex (woman/man), prevalence of T2D (yes/no), age (years), BMI (kg/m^2^), smoking status (never/current/former), physical activity (met/day), educational level (illiterate/primary education, secondary education, and academic/graduate), use of insulin (yes or no), use of oral antidiabetic drugs (yes or no), use of oral anticoagulants drugs (yes or no), use of vitamin D supplements (yes or no), and use of estrogens (yes or no), recruiting center, and total energy intake (kcal/day)

The distribution of total low BMD status was unlike between BMD measures. The first tertile of TF, TR, and LS had 71 (3 osteoporosis cases and 68 osteopenia cases), 54 (1 osteoporosis case and 53 osteopenia cases), and 99 (13 osteoporosis cases and 86 osteopenia cases) events, respectively. While the third tertile of TF, TR, and LS had 97 (1 osteoporosis case and 96 osteopenia cases), 86 (2 osteoporosis cases and 84 osteopenia cases), and 119 (26 osteoporosis cases and 93 osteopenia cases) events, respectively. The odds ratios (OR) and 95% CI for low BMD status across tertiles of the M-DIS score are shown in Table 3. The M-DIS score was associated with low BMD status in both the TF (OR 1.71, 95% CI 1.12–2.64; *P* trend: 0.015) and the TR sites (OR 2.02, 95% CI 1.29–3.21; *P* trend: 0.002) when comparing those participants in the third tertile versus those in the first tertile. Those participants in the top M-DIS tertiles had a non-significant increased odds of low BMD status compared to those in the reference tertile (OR 1.44, 95% CI 0.95–2.17, *P* trend 0.092).

**Table 3:** Odds ratio and 95% confidence intervals between low BMD and tertiles of M-DIS

	Tertile 1	Tertile 2	Tertile 3	*P* for trend
Total femur, *n*	362	361	361	
Low BMD, % (*n*)	19.60 (71)	24.90 (90)	26.90 (97)	
Crude model	1 (reference)	1.36 (0.96, 1.94)	1.51 (1.06, 2.14)	0.022
Adjusted model*	1 (reference)	1.44 (0.99, 2.13)	1.71 (1.12, 2.64)	0.015
Trochanter, *n*	377	376	376	
Low BMD, % (*n*)	13.50 (54)	19.70 (74)	22.90 (86)	
Crude model	1 (reference)	1.47 (1.00, 2.15)	1.78 (1.22, 2.60)	0.003
Adjusted model*	1 (reference)	1.57 (1.04, 2.40)	2.02 (1.29, 3.21)	0.003
Lumbar spine L1–L4, *n*	324	323	323	
Low BMD, % (*n*)	30.60 (99)	36.50 (118)	36.80 (119)	
Crude model	1 (reference)	1.31 (0.94, 1.82)	1.33 (0.96, 1.84)	0.093
Adjusted model*	1 (reference)	1.43 (0.99, 2.08)	1.44 (0.95, 2.17)	0.092

Logistical regression models were used to evaluate the association between low BMD and tertiles of the dietary inflammatory score. Results are expressed as odds ratio (95% CIs), and % (*n*). BMD was considered low for all participants with a *T* score less than − 1 in every BMD measure

*M-DIS* modified dietary inflammatory score

*BMD* bone mineral density

^*^ Models adjusted for sex (woman/man), prevalence of T2D (yes/no), age (years), BMI (kg/m^2^), smoking status (never/current/former), physical activity (met/day), educational level (illiterate/primary education, secondary education, and academic/graduate), use of insulin (yes or no), use of oral antidiabetic drugs (yes or no), use of oral anticoagulants drugs (yes or no), use of vitamin D supplements (yes or no), and use of estrogens (yes or no), recruiting center and total energy intake (kcal/day)

**Fig. 1:**
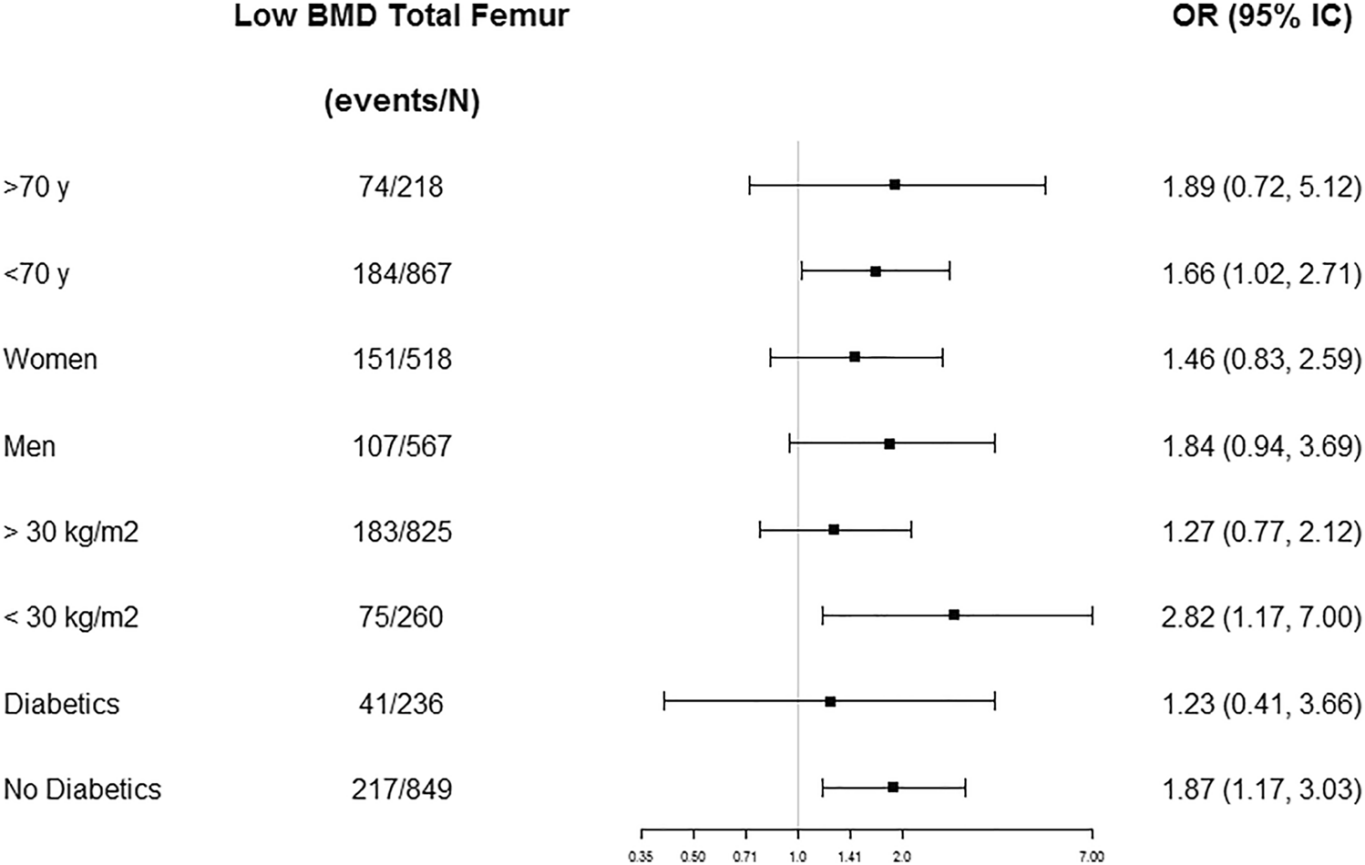
Odds ratios (ORs) and 95% confidence intervals (95% CI) for low BMD in total femur comparing tertile 3 versus tertile 1 of the M-DIS score stratified by age, sex, BMI, and T2D status. ORs were adjusted by sex, the prevalence of T2D, age (years), BMI (m/kg^2^), smoking, education, recruiting center, physical activity (METs/day), use of insulin (yes or no), use of oral antidiabetic drugs (yes or no), use of oral anticoagulants drugs (yes or no), use of vitamin D supplements (yes or no), use of estrogens (yes or no), and energy intake (kcal/day) excluding age, sex, BMI, or T2D status when it is stratified by one of these variables

**Fig. 2:**
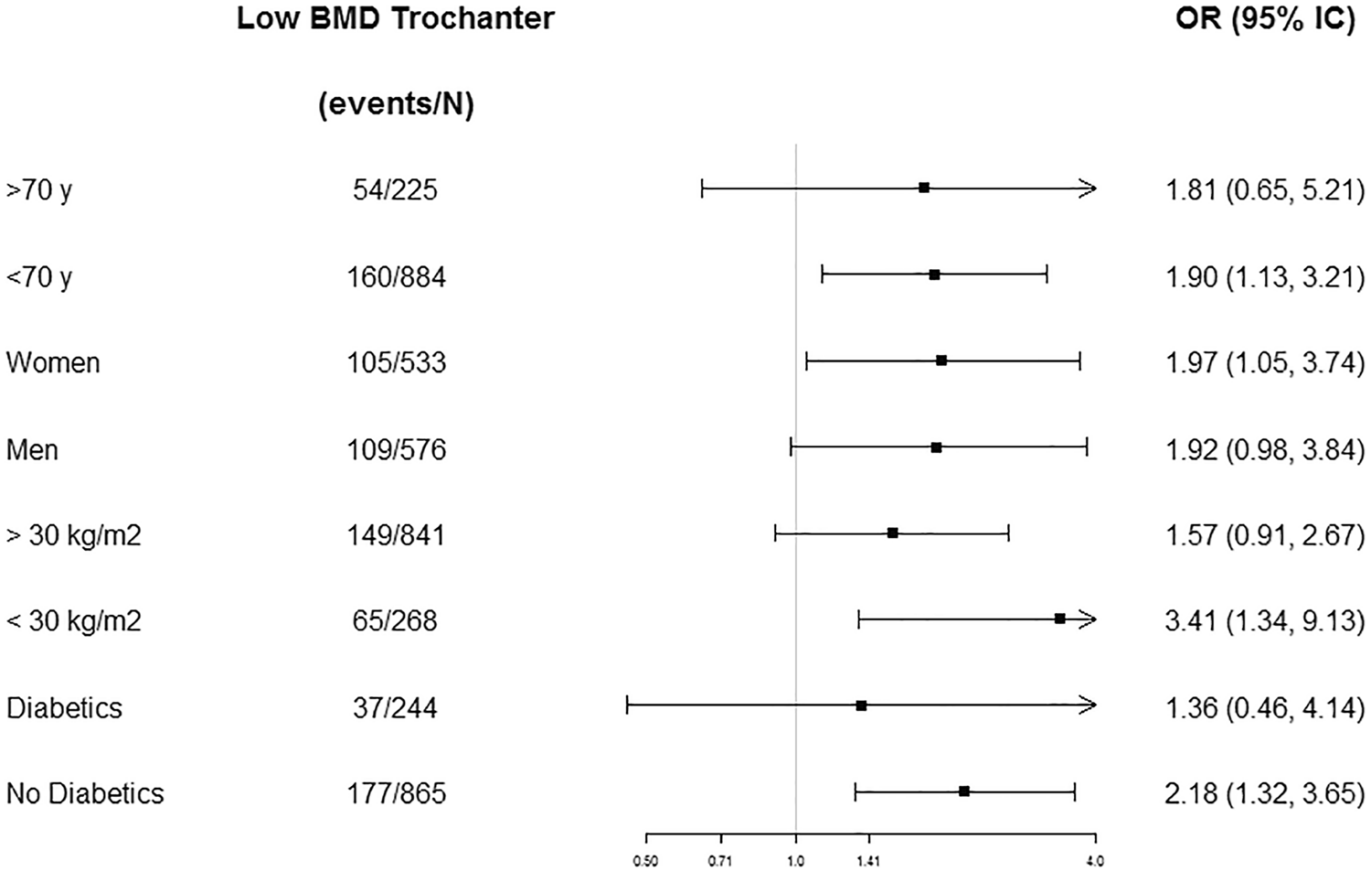
Odds ratios (ORs) and 95% confidence intervals (95% CI) for low BMD in trochanter comparing tertile 3 versus tertile 1 of the M-DIS score stratified by age, sex, BMI, and T2D status. ORs were adjusted by sex, the prevalence of T2D, age (years), BMI (m/kg^2^), smoking, education, recruiting center, physical activity (METs/day), use of insulin (yes or no), use of oral antidiabetic drugs (yes or no), use of oral anticoagulants drugs (yes or no), use of vitamin D supplements (yes or no), use of estrogens (yes or no), and energy intake (kcal/day) excluding age, sex, BMI, or T2D status when it is stratified by one of these variables

**Fig. 3:**
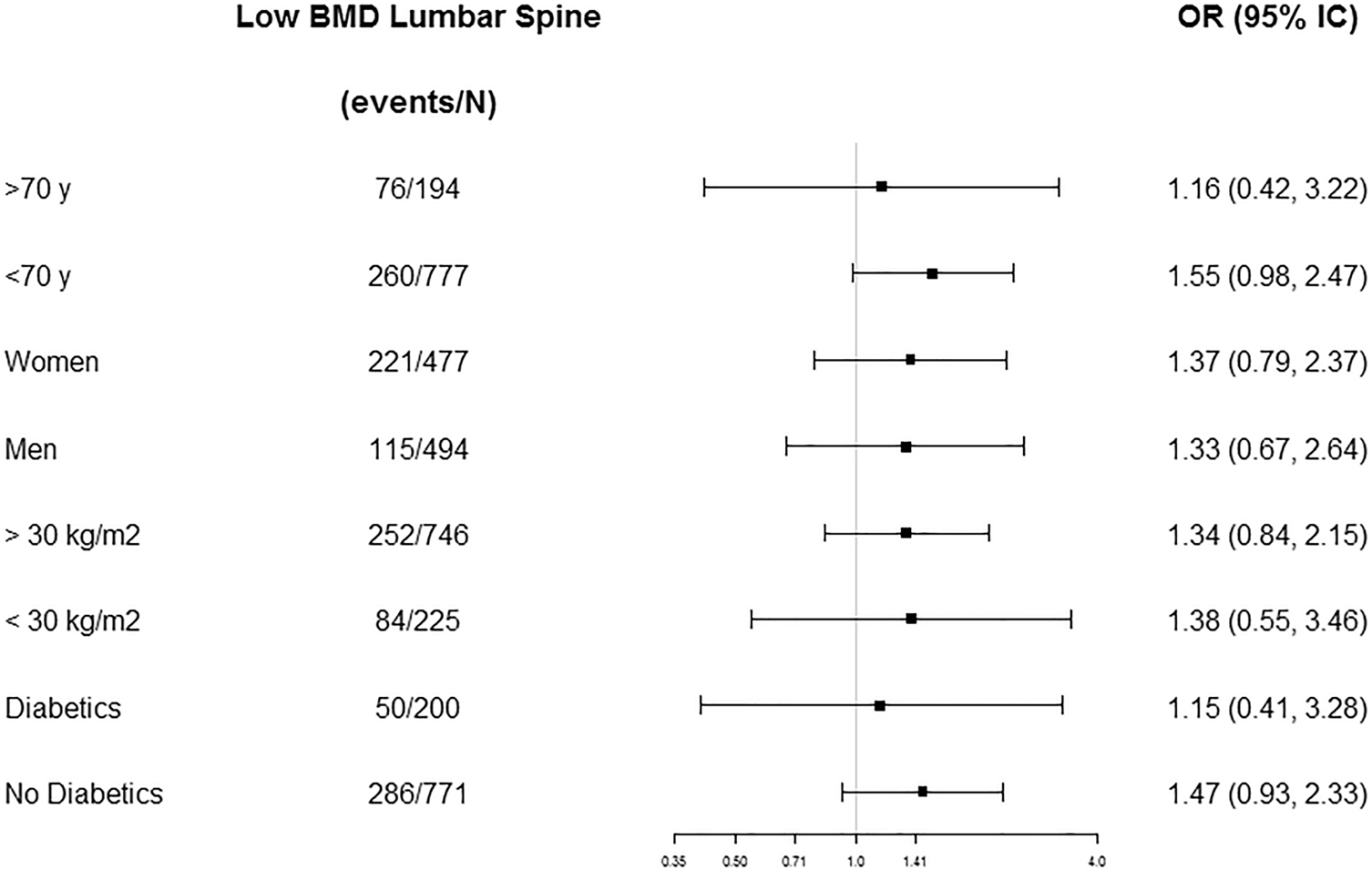
Odds ratios (ORs) and 95% confidence intervals (95% CI) for low BMD in lumbar spine comparing tertile 3 versus tertile 1 of the M-DIS score stratified by age, sex, BMI, and T2D status. ORs were adjusted by sex, the prevalence of T2D, age (years), BMI (m/kg^2^), smoking, education, recruiting center, physical activity (METs/day), use of insulin (yes or no), use of oral antidiabetic drugs (yes or no), use of oral anticoagulants drugs (yes or no), use of vitamin D supplements (yes or no), use of estrogens (yes or no), and energy intake (kcal/day) excluding age, sex, BMI, or T2D status when it is stratified by one of these variables

Results from the sensitivity analyses did not differ from the general ones (Figs. 1, 2 and 3). BMD at the TF showed a significant association between M-DIS's tertiles and low BMD status, when participants were younger than 70 years (OR 1.66, 95% CI 1.02–2.71), presented overweight (OR 2.82, 95% CI 1.17–7.00) and were no diabetics (OR 1.87, 95% CI 1.17–3.03) when comparing those participants in the third versus those in the first tertile. Similarly, BMD at the TR showed significant associations between M-DIS’s tertiles and low BMD status when participants were younger than 70 years (OR 1.90, 95% CI 1.13–3.21), were women (OR 1.97, 95% IC 1.05–3.74), presented overweight (OR 3.41, 95% IC 1.34–9.13), and do not have diabetes (OR 2.18, 95% IC 1.32–3.65). No significant differences in the associations between M-DIS tertiles and low BMD status were found in LS.

## Discussion

In the current cross-sectional analysis, we reported a significant association between a high M-DIS score and a low bone mineral density at different sites (total femur, trochanter, and lumbar spine), along with an increased risk of osteopenia or osteoporosis, in a senior Mediterranean population with metabolic syndrome. Likewise, we observed an inverse association between the risk of osteopenia/osteoporosis and M-DIS in younger participants, without diabetes and with overweight in the femur areas. These results suggest that a pro-inflammatory diet favors an adverse bone environment that promotes bone loss mechanisms.

The M-DIS has been introduced as a tool to link individuals' food intakes with the overall inflammatory potential of their diets [14]. Several epidemiological trials have already investigated the relationship between the M-DIS score and bone health, but up to now, none have assessed the effect of M-DIS in a population with high adherence to the MedDiet (which is already recognized for its anti-inflammatory effect), and presenting with other comorbidities associated with a pro-inflammation state. In our study, the M-DIS score was inversely associated with BMD in both the femur (total femur and trochanter) and lumbar spine (lumbar spine L1–L4) when we used a pooled sample of women and men. A similar trend was observed when we stratified the analysis by sex. These results are consistent with previous studies. In a recent Korean study that included 2778 elderly postmenopausal women aged > 50 years [31], a higher M-DIS score was associated with low femoral BMD. In the Women’s Health Initiative study, lower hip BMD was observed in postmenopausal women with a lower M-DIS score compared to women with a high M-DIS score at baseline, although lower BMD losses were observed after 6 years of follow-up suggesting a possible positive effect of an anti-inflammatory diet [32]. Similarly, in the context of the NHANES (United States National Health and Nutrition Examination Survey Study), the authors reported that an increased M-DIS score was associated with a decreased BMD across most of the measured bone sites (total femur, femoral neck, trochanter, intertrochanter, Wards triangle, total spine, lumbar vertebrae L3, and lumbar vertebrae L4) in both, men and women [33].

Inflammation has been previously associated with bone health and osteoporosis. It is known that inflammatory cytokines can mediate bone loss via stimulation of osteoclast formation and promotion of the OPG/RANK/RANKL pathway [10, 11, 34]. Actually, high serum concentrations of the inflammatory cytokines IL-6 and TNF-α have been associated with osteoporosis [7, 9, 11]. Furthermore, in vitro studies have shown that these molecules are prone to influence osteoclasts by stimulating bone resorption [35–38].

Diet quality is a modifiable lifestyle factor that can affect bone metabolism [21, 39, 40]. Several studies have assessed the association between the M-DIS score with numerous inflammatory biomarkers [20, 41]. The consumption of some nutrients and dietary patterns considered to be healthy have been associated with better concentrations of cytokines and specific anti-inflammatory bone biomarkers [12, 42, 43], while others, such as carbohydrates and saturated fatty acids, with a pro-inflammatory coefficient in the M-DIS score, have been related to a poorer bone status, osteoporosis development and fractures risk [44]. In our study, people in the first tertile of M-DIS were shown to have higher macronutrients and total energy intake compared to participants in the third tertile. Although these macronutrients are associated with M-DIS pro-inflammatory values, individuals in this category were also observed to have a higher intake of anti-inflammatory micronutrients that could potentially counteract the increased pro-inflammatory compounds. This might contribute to a protective effect on bone metabolism related to a high nutritionally dense diet.

In addition, participants in the highest tertile of M-DIS presented an increased risk of low BMD status (i.e., participants with a higher risk of presenting with osteopenia or osteoporosis) in the femur but not in the lumbar spine. Similarly, in two studies conducted in Korean populations, a high M-DIS score was significantly related to higher ORs for low BMD status (osteopenia cases + osteoporosis cases) in the total femur and femoral neck but not in the lumbar spine [31, 45]. These discrepancies regarding the affected area could be partly explained due to differences in the relationship between BMD with bone mineral content (BMC) and bone area size (BAS) which modify the risk of developing osteoporosis in the spine and hip [46]. It could be speculated that inflammation produces greater effects on larger bones than in smaller bones and, therefore, anti-inflammatory-property diets or diets composed of a large proportion of anti-inflammatory compounds (i.e., diets with negative values of M-DIS) might produce more benefits in terms of bone health for the hip area than the lumbar spine. Additionally, in stratified analyses by T2D, BMI, sex, and age, younger participants with overweight and without diabetes showed an inverse association between M-DIS and the risk of osteopenia/osteoporosis. In this regard, adequately controlled T2D has shown a positive effect on bone health and helped to reverse bone impairments [47, 48], which might partially explain the observed differences between groups. Although we adjusted our models by medication (including diabetic medication), we cannot discard that some of our overweight participants were in a pre-diabetes status without medication that increased the risk of bone damage.

There are other potential mechanisms associated with inflammation that can be boosted by the inflammatory effect of a diet that can be related to bone metabolism. Pro-inflammatory diets might promote glucose and insulin secretion homeostasis disruption, and assist in the chronic activation of pro-inflammatory pathways that benefit the osteoclastogenesis, bone resorption processes, and the disruption of the normal function of osteoblasts [49–51]. In the same way, oxidative stress, as an exacerbating circumstance of the inflammatory process, may also have a key role in bone deterioration [52]. High concentrations of reactive oxygen species and reactive nitrogen species derived from the cellular redox process can reduce bone mass and increase apoptosis of osteoblasts by inhibiting osteoblastogenesis and stimulating RANKL and TNF-α secretion [53, 54]. Finally, the antioxidant abilities of processes in the body decline with age, but anti-inflammatory diets with a high quantity of anti-oxidative compounds may help to balance these processes. In accordance with previous investigations, our findings increase the evidence regarding the potential ability of improved diet quality as measured with the M-DIS might help to modulate the inflammatory processes and decrease the risk of bone disease.

Certain limitations of our study are required to be mentioned. First, we cannot establish a cause–effect relation because of the cross-sectional nature of the analyses, and inference between BMD and the M-DIS score is limited. Additionally, we cannot discard a possible role of secondary factors (such as physical activity) despite the adjustment by potential confounders. Second, this study was carried out in an elderly Mediterranean population with metabolic syndrome, and our results cannot be generalized to other populations. Furthermore, we cannot rule out the possibility that low M-DIS (anti-inflammatory diet) values produce enough protective effect on BMD in a population with high adherence to the MedDiet. Additionally, the process of collecting dietary data and computing the M-DIS score without some items of the original score impossible to estimate from FFQs could induce some degree of misclassification (measurement error). This potential measurement error would be likely non-differential because of the prospective design of our study and would address the probable bias of the results towards the null. However, it is known that the use of validated FFQ is a correct methodology in a large perspective [55], especially when trained dietitians assisted the participants to properly complete the FFQ. Finally, the lack of inflammatory markers in our study does not enable us to confirm any potential mechanism that might explain the observed results. However, the major strengths of the study are the use of a DXA scan for measuring BMD, the control for many potential confounding variables, and the inclusion of sensitivity analyses with similar trends.

## Conclusions

In conclusion, the results of our study suggest that a high pro-inflammatory diet, measured with an M-DIS score, is associated with lower BMD in a Mediterranean population with metabolic syndrome. Further studies are needed to clarify the potential mechanism that could explain the observed associations.

## Supplementary Information

Below is the link to the electronic supplementary material.

Supplementary file1 (DOCX 33 KB)
