# Supplementary material for: Inflammatory potential of diet and bone mineral density in a senior Mediterranean population: a cross-sectional analysis of PREDIMED-Plus study
Source: Eur J Nutr. 2021 Nov 29;61(3):1445–55. doi: 10.1007/s00394-021-02751-5 (PMC8921075; doi:10.1007/s00394-021-02751-5)

Supplemental table 1. Nutrient and food daily consumption according to tertiles (T) of the modified dietary inflammatory score in the subsample of PREDIMED Plus study.

|  | T1 (n = 368)  (most anti-inflammatory) | T2 (n = 368) | T3 (n = 368)  (most pro-inflammatory) |
| --- | --- | --- | --- |
| Caffeine (g) | 25.72 (25.22) | 24.06 (25.62) | 30.14 (31.73) |
| Alcohol (g) | 14.89 (17.90) | 11.49 (15.71) | 8.41 (11.83) |
| Vitamin B12 (μg) | 13.04 (5.56) | 9.98 (3.61) | 7.71 (2.92) |
| Vitamin B6 (mg) | 2.90 (0.47) | 2.32 (0.34) | 1.89 (0.35) |
| Carbohydrate (g) | 290.35 (75.98) | 247.08 (67.80) | 212.15 (61.97) |
| Cholesterol (mg) | 444.69 (126.10) | 393.11 (108.79) | 342.82 (102.75) |
| Energy (kcal) | 2839.49 (555.77) | 2450.49 (514.44) | 2108.55 (473.23) |
| Total fat (g) | 123.08 (29.63) | 109.81 (26.52) | 95.57 (25.53) |
| Fiber (g) | 34.15 (7.82) | 26.13 (5.14) | 19.54 (4.77) |
| Folic acid (μg) | 448.26 (85.00) | 347.34 (57.48) | 267.82 (57.60) |
| Garlic (g) | 1.71 (1.22) | 1.36 (0.93) | 0.98 (0.85) |
| Fe (mg) | 20.29 (3.30) | 16.48 (2.33) | 13.36 (2.45) |
| Mg (mg) | 521.14 (91.71) | 413.69 (65.23) | 332.10 (62.28) |
| MUFA (g) | 62.77 (16.50) | 57.32 (14.20) | 50.06 (13.88) |
| Vitamin B3 (mg) | 49.11 (8.61) | 40.81 (7.48) | 34.83 (7.35) |
| n-3 Fatty acids (g) | 1.15 (0.48) | 0.89 (0.45) | 0.66 (0.34) |
| n-6 Fatty acids (g) | 17.86 (7.37) | 14.43 (5.30) | 11.19 (4.00) |
| Onion (g) | 27.14 (14.68) | 22.57 (12.09) | 16.72 (10.81) |
| Protein (g) | 116.54 (20.72) | 98.37 (19.13) | 85.24 (18.12) |
| PUFA (g) | 21.86 (7.91) | 17.47 (5.48) | 13.68 (4.57) |
| Vitamin B2 (mg) | 2.49 (0.68) | 1.95 (0.49) | 1.67 (0.49) |
| SFA (g) | 30.79 (9.38) | 27.60 (8.67) | 24.76 (8.17) |
| Se (μg) | 142.59 (32.82) | 120.05 (28.69) | 99.42 (25.68) |
| Vitamin B1 (mg) | 2.03 (0.36) | 1.65 (0.27) | 1.36 (0.29) |
| Trans fat (g) | 0.67 (0.40) | 0.63 (0.41) | 0.58 (0.37) |
| Vitamin A (RE) | 1515.24 (825.52) | 1044.19 (444.56) | 730.74 (342.38) |
| Vitamin C (mg) | 276.76 (90.07) | 201.77 (64.74) | 143.18 (48.78) |
| Vitamin D (μg) | 7.87 (3.50) | 6.12 (3.15) | 4.42 (2.41) |
| Vitamin E (mg) | 14.27 (5.42) | 10.88 (3.13) | 8.27 (2.11) |
| Zn (mg) | 16.13 (3.13) | 13.43 (2.66) | 11.38 (2.58) |
| Tea (g) | 16.69 (34.62) | 8.87 (24.01) | 9.54 (25.53) |
| Beta carotene (μg) | 9091.45 (4953.12) | 6265.13 (2667.39) | 4384.45 (2054.30) |
| Values are presented as mean (SD). | | | |

Supplemental figure 1. Scoring of each food items used to calculate M-DIS.


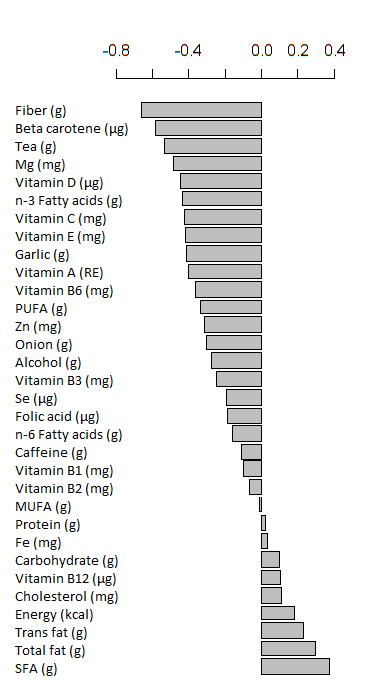

Supplement: Supplementary file 1 — Supplementary file1 (DOCX 33 KB) [file 394_2021_2751_MOESM1_ESM.docx]
